# Primary synovial sarcoma of the shoulder: Case report of the “triple sign” on proton density magnetic resonance imaging

**DOI:** 10.1016/j.radcr.2022.11.077

**Published:** 2022-12-26

**Authors:** Aleksandar A. Georgiev, Desislava Tashkova, Lyubomir Chervenkov, Vania Anastasova, Tanya Kitova

**Affiliations:** a Department of Diagnostic Imaging, Medical University Plovdiv, Bul. Vasil Aprilov 15A, Plovdiv, 4002, Bulgaria; b Department of Pathoanatomy, Medical University Plovdiv, Bul. Vasil Aprilov 15A, Plovdiv, 4002, Bulgaria; c Department of Propaedeutics of Surgical Diseases, Section of Plastic, Reconstructive and Aesthetic Surgery and Thermal Trauma, Medical University Plovdiv, Bul. Vasil Aprilov 15A, Plovdiv, 4002, Bulgaria; d Department of Human Anatomy, Histology and Embryology, Medical University Plovdiv, Bul. Vasil Aprilov 15A, Plovdiv, 4002, Bulgaria; e Medical College, Trakia University, Stara Zagora, 6015, Bulgaria

**Keywords:** Oncology, MRI, PD, Synovial neoplasm, Rare, MR, magnetic resonance, MRI, magnetic resonance imaging, SS, synovial sarcoma, PD, proton density, FS, fat-suppressed, TSE, turbo spin-echo, TR, repetition time, TE, time to echo

## Abstract

The first case of synovial sarcoma was published in 1893. The disease is a type of primary malignancy of the soft tissues. It is a rare and aggressive neoplasm of unknown tissue origin, characterized by strong metastatic potential and poor prognosis. The present case of a 64-year-old male patient with pain and swelling in his right shoulder and progressive loss of movement demonstrates an uncommon location for the neoplasm. Magnetic resonance proton-density fat-suppressed turbo spin-echo sequences show a heterogeneous mass in the right shoulder. The lack of homogeneity in the signal has been described in medical literature as the “triple sign” and is represented by low, intermediate, and high signal intensity areas through the neoplasm. Visible serpentine vessels spread through the tumor. There was a visible metastatic disease in the regional lymph nodes and metastatic foci in the adjacent bones. Pathological analysis of the tumor confirmed the diagnosis of biphasic synovial sarcoma. An oncological committee advised chemotherapy and radiotherapy. More prominent magnetic resonance imaging findings in synovial sarcoma that may facilitate the diagnostic process are the inhomogeneity and “triple sign” in proton density and T2 sequences, multilobulated tumors, septa, irregular borders, serpentine vascular channels, engagement of the adjacent bones and bone marrow, and involvement of the joint synovia.

## Introduction

Primary synovial sarcoma (SS) was initially described in medical literature in 1893 and represents a malignancy of the soft tissues [1], [2], [3], [4], [5], [6], [7]. Synovial sarcoma of the shoulder accounts for 6% of all synovial sarcomas [1], [2], [3], [4], [5], [6]. Some of the names used to describe this lesion include tendosynovial sarcoma, synovial endothelioma, malignant synovioma, synovioblastic sarcoma, synovial cell sarcoma, and synovioma [2], [3], [4], [5], [6], [7]. Contrary to its name, the disease usually does not arise from inside the joint but from the surrounding soft tissues. Synovial sarcomas primarily affect adolescents and young adults. The knee is the primary location of SS within the popliteal fossa [4]. Synovial sarcoma is a neoplasm with extensive metastatic potential. Due to the aggressiveness of SS, pathological and imaging assessments are essential for staging and visualizing the extent of the lesion to guide the therapeutic process.

## Case report

The presented uncommon case was of a 64-year-old male patient with pain and swelling in his right shoulder. The pain started several months before the imaging examination. The patient did not recall any accident or trauma. The patient was not vaccinated against COVID-19. Initially, he took pain relief medications with little to no effect. He tried immobilizing the shoulder and some alternative medicines with no result, such as herbal compress. Around 2 months after the initial complaints, the patient experienced swelling in his right shoulder, which began to grow. A few weeks later the soft-tissue mass almost doubled in size, he could no longer raise his arm above the shoulder. The patient consulted an orthopedist and was directed to the Department of Oncological Imaging. A magnetic resonance imaging (MRI) of the right shoulder was performed. Before scanning, the patient's arm was almost immobile during the physical examination, and there was a large soft tissue mass in the right shoulder. The tumor was red to purplish in some regions, rigid, and uneven on palpation. During the MRI, the patient experienced severe pain. Although pain relief medication was introduced before the scan, some of the images have motion artifacts due to uncontrollable shaking of the limb during the image acquisition phases.

**Fig. 1:**
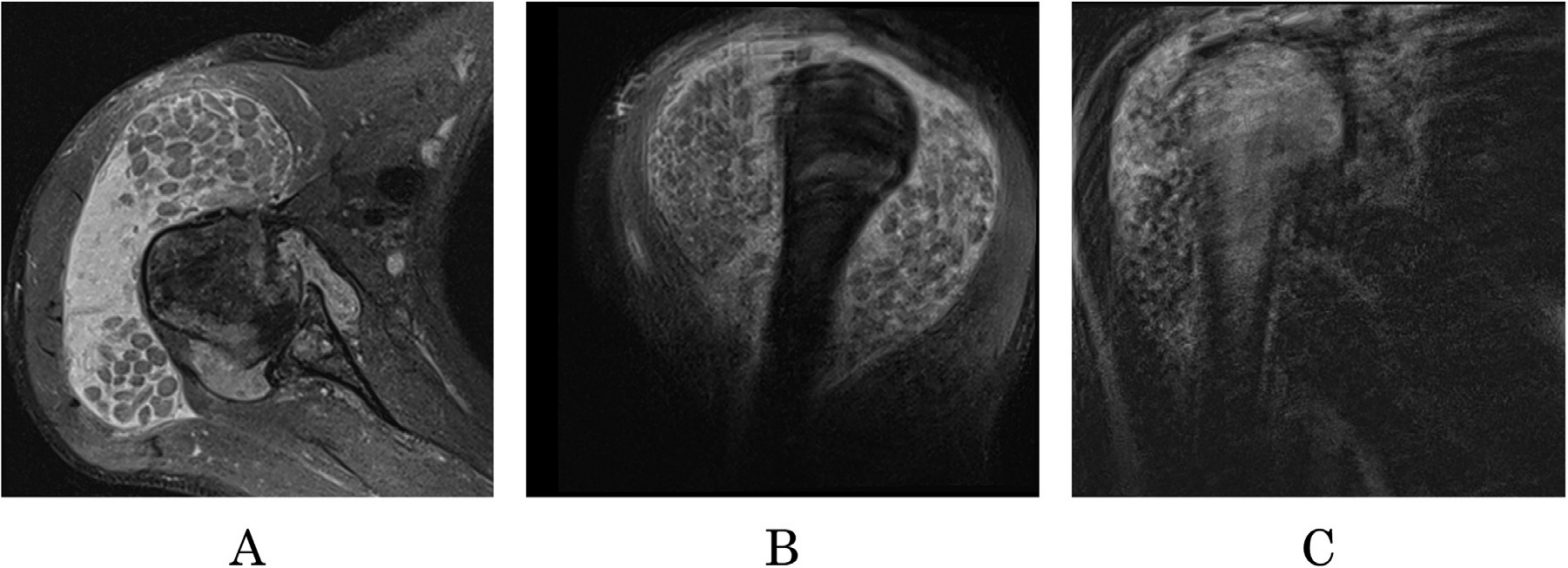
In Figure 1A, an axial view of PD_FS_TSE sequences shows a tumor formation in the right shoulder. In 2A, a coronal view of the SS in PD MRI. 2C, the PD_FS_TSE shows the sagittal view of the neoplasm.

Proton-density fat-suppressed turbo spin-echo (PD_FS_TSE) MRI sequences show heterogeneous formation in the right shoulder (Figs. 1A-C). (See Video 1, Supplemental Video, which demonstrates the PD_FS_TSE appearance of synovial sarcoma of the right shoulder in the axial plane; see Video 2, Supplemental Video, which demonstrates the PD_FS_TSE appearance of synovial sarcoma of the right shoulder in the sagittal plane; see Video 3, Supplemental Video, which demonstrates the PD_FS_TSE appearance of synovial sarcoma of the right shoulder in the coronal plane.)

The predominant high signal intensity with visible heterogeneity is a crucial sign of synovial sarcoma. Heterogeneity is described in medical literature as the “triple sign.” The neoplasm is multifocal and has an irregular shape and visible septa. In some regions, the outline of the neo-plasm appeared to be irregular. Scattered, inhomogeneous, low-intensity nodules are visible inside the tumor. The soft tissue mass invaded the joint capsule and grew inside the joint gap. Neovascularization is present, and visible snake-like vessels spread through the tumor. There was a visible metastatic disease in the regional lymph nodes and metastatic foci in the adjacent bones: the humerus and right scapula.

**Fig. 2:**
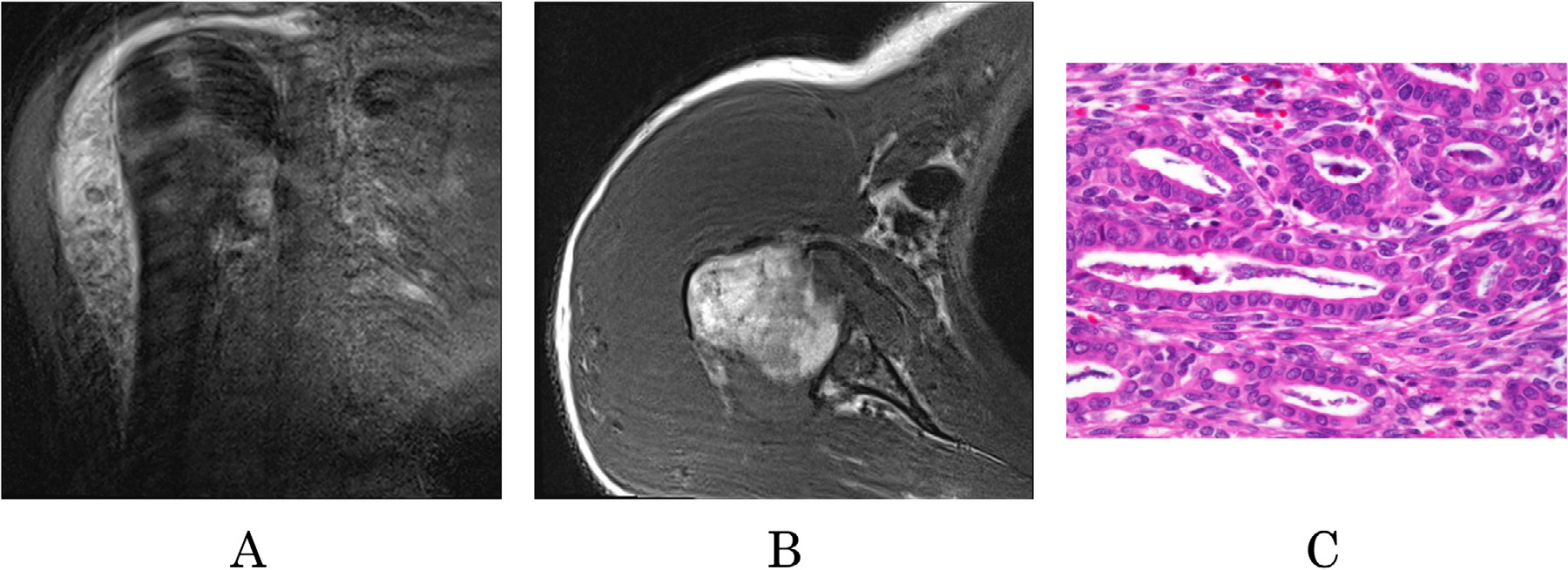
In 2A, T2_FS_TSE demonstrates the sagittal view of the SS. In 2B T1, the axial view reveals similar signal intensity to surrounding tissues. In 2C Hematoxylin and eosin stain of the synovial sarcoma.

The same mass on T2 TSE MRI is visible in Fig. 2A. (See Video 4, Supplemental Video, which demonstrates the T2_TSE appearance of synovial sarcoma of the right shoulder in the coronal plane.)

Similar to PD MR images in T2, the tumor appears heterogeneous with predominantly high signal intensity, and snake-like vessels were also observed. The neoplasm is multifocal and has an irregular shape and visible septa. Lymph node invasion and metastatic bone disease are present. An almost identical finding is seen in the fat-suppressed T2 series.

The T1 MR images are presented in Fig. 2B. (See Video 5, Supplemental Video, which demonstrates the T1_TSE appearance of synovial sarcoma of the right shoulder in the axial plane.) In T1, the neoplasm appears multifocal with a visibly irregular shape and neovascularization. The signal intensity was very similar to or slightly lower than the surrounding muscle tissue. The septa were not well demarcated. Lymph node invasion and metastatic bone disease were visible in the images.

The suspected diagnosis based on the MRI findings was synovial sarcoma. The differential diagnosis could be malignant fibrous histiocytoma and other soft-tissue neoplasms. Pathological verification of the tumor showed a biphasic SS with translocation of t(X;18) chromosome (p11.2; q11.2), which validates the suspected MRI diagnosis of SS (Fig. 2C).

Because of the size and intraarticular location of the neoplasm and the presence of metastatic disease, the treatment plan for the patient was decided by a multidisciplinary oncology committee. The decision was to perform “sandwich” therapy initiated with 3 courses of chemotherapy followed by radiotherapy and another 4 courses of chemotherapy. Before the last chemotherapy course, a staging CT scan and MRI of the shoulder will be performed. Depending on the response to the treatment, surgical excision of the prime tumor could be planned.

## Discussion

Synovial sarcoma is a rare neoplasm with a poor long-term prognosis. It is an aggressive neoplasm that is characterized by monophasic or biphasic differentiation. A pathognomonic sign is a translocation of the t(X;18) chromosome (p11.2; q11.2) to create and express the SYT-SSX fused gene [1], [2], [3], [4], [5], [6]. Synovial sarcoma is affecting the shoulder and arises in less than 6% of the cases [1,4]. An intraarticular SS usually develops in the frontal part of the knee joint (fat pad of Hoffa) [4]. MRI has a superior soft-tissue resolution and is considered the “gold” imaging modality for evaluating the characteristics and extent of SS [7], [8], [9], [10], [11], [12], [13]. Typically, synovial sarcoma appears as an inhomogeneous multilobulated soft-tissue tumor on T1-weighted sequences, with signal intensity similar to or slightly higher than normal muscles [14,15]. The predominant high signal intensity with visible heterogeneity is also a centerpiece feature of SS on T2-weighted MRI [13-16]. This nonhomogeneity in the signal has been described as the “triple sign” [15], represented by low, intermediate, and high signal intensity areas. As the presented case demonstrates in PD imaging, heterogeneity with predominant high signal intensity is also a key sign. Heterogeneity is visible inside the low signal intensity foci on PD sequences. The visible lack of homogeneity and presence of the “triple sign” in PD and T2 MRI is possibly resulting from solid parts of the neoplasm mixed with necrosis or hemorrhagic foci. Connective tissue (fibrosis) and calcifications remain with low MRI signal intensity [4,15]. The “triple sign” can be seen in around 35%-57% of SS cases, and more commonly in larger tumors [1,15]. We have to keep in mind that the “triple sign” is also an imaging feature of other soft-tissue neoplasms. One such malignancy is fibrous histiocytoma. The “triple sign” is not very specific for SS diagnosis. A multilobulated presentation of the neoplasm and finding of septations, as visible in the presented case, is reported in 67%-75% of synovial sarcoma cases [13,14]. The septa are better visualized on PD and T2-weighted images. Areas of liquid collections and high signal intensity zones in T1 and T2 MRI sequences are common and are visible in up to 47% of SS neoplasms. In PD imaging denser tissues (those with high proton density) appear with high signals in MRI. PD-weighted sequence creates contrast generally by reducing the effect of T1 and T2 differences with longer repetition times (2000-5000 ms) and shorter time to echo (10-20). Fluid levels have been reported in 10%-25% of synovial sarcomas cases [1,^4^,^10^,11]. In the presented case, cystic areas and hemorrhagic foci are visible in the joint space. Smaller than 5 cm lesion may be visible as a non-homogeneous focus on all MR sequences and may suggest a neoplasm with a better prognosis or smaller metastatic potential [12], [13], [14]. The value of MRI clinical features for synovial sarcoma is described by Ashikyan and co-workers [8]. An important visual characteristic that indicates a high-grade SS includes the “triple sign,” absence of calcification, cystic components, and bleeding [1,^4^,12]. Patients with calcified SS, lack of visible hemorrhage, or absence of the “triple sign” have a statistically significant likelihood of disease-free survival. The presence of the “triple sign” may help understand the complex multitude of parameters associated with synovial sarcomas. The long-term prognosis in SS depends on the size of the neoplasm, the location, and the possibility of a clean surgical margin [7,9]. Verbeek et al. found a more favorable prognosis for SS of the shoulder compared to primary locations in the body [1]. The course of primary SS in the shoulder is more on par with that of the limbs compared to that of the chest, abdomen, or pelvic primary neoplasm.

## Conclusion

In conclusion, we can say that the most common MRI findings in synovial sarcoma are the inhomogeneity and “triple sign” in PD and T2 sequences, the presence of multilobulated neoplasm, septa, serpentine vascular channels, affecting the adjacent bones, and bone marrow, and involvement of the joint synovia. T1 sequences were not indicative of synovial sarcoma.

## Patient consent

Written patient consent for publication has been obtained.

## Ethical compliance

All procedures performed in studies involving human participants were in accordance with the ethical standards of the institutional research committee of Medical University Plovdiv number 132/01.02.2022, and with the 1964 Helsinki Declaration and its later amendments or comparable ethical standards.

## Author contributions

A.G. is responsible for the conception of the work, data collection, data analysis, and interpretation. A.G is also responsible for drafting the article. D.T. and V.A. are responsible for data analysis. L.C. and T.K. are responsible for critical revision of the article and final approval of the version to be published.

## Data access statement

All research data supporting this publication is included in the article.

## Appendix Supplementary materials

Image, video 1

Image, video 2

Image, video 3

Image, video 4

Image, video 5
